# BD Vaginal Panel assay results on the high-throughput BD COR system compared to the BD MAX system

**DOI:** 10.1128/spectrum.00235-24

**Published:** 2024-06-20

**Authors:** Elizabeth Stonebraker, Wallace Greene, Stephanie N. Taylor, Catherine L. Cammarata, April Bobenchik, Elizabeth Lockamy

**Affiliations:** 1 Becton, Dickinson and Company, BD Life Sciences–Diagnostic Solutions, Sparks, Maryland, USA; 2 Department of Pathology and Laboratory Medicine, Milton S. Hershey Medical Center, Penn State College of Medicine, Hershey, Pennsylvania, USA; 3 Department of Medicine, Section of Infectious Diseases, Louisiana State University Health Sciences Center, New Orleans, Louisiana, USA; Johns Hopkins University, Baltimore, Maryland, USA

**Keywords:** vaginitis, sexually transmitted diseases, molecular diagnostic techniques, diagnostic accuracy, high-throughput screening assays

## Abstract

Molecular-based assays demonstrate excellent sensitivity for the detection of vaginitis causes. Here, the high-throughput BD Vaginal Panel for BD COR System (VP-COR) performance was compared to that of the predicate, BD MAX Vaginal Panel for BD MAX System (VP-MAX). Clinical or contrived samples were used to determine the agreement between VP-COR and VP-MAX. Acceptance criteria for VP-COR agreement were as follows: bacterial vaginosis (BV) required a positive percent agreement (PPA) point estimate of ≥95% and a negative percent agreement (NPA) point estimate of ≥98%; *Candida* group, *Candida glabrata*, *Candida krusei*, and *Trichomonas vaginalis* (TV) required a PPA and NPA point estimate of ≥95% [with lower bound of 95% confidence interval (95% CI) ≥90%]. PPA was 99.5% (95% CI: 97.5–100) and 97.9% (95% CI: 96.5–98.8) for BV contrived (*n* = 516) and BV clinical (*n* = 1,050) specimens, respectively. For the *Candida* group (clinical; *n* = 724), *C. glabrata* (contrived; *n* = 544), *C. krusei* (contrived; *n* = 522), and TV (clinical; *n* = 702), PPA was 99.4% (95% CI: 98.0–99.9), 100% (95% CI: 97.9–100), 100% (95% CI: 97.6–100), and 99.7% (95% CI: 98.3–100), respectively; the lowest lower bound CI value was 97.6%. NPA was >95% for BV contrived and BV clinical specimens. For the *Candida* group, *C. glabrata*, *C. krusei*, and TV, NPA was ≥98.9%; the lowest lower bound CI value was 97.3%. These results demonstrate the equivalent performance of the VP-COR assay when compared to VP-MAX.

**IMPORTANCE:**

Vaginitis is common among women of reproductive age, resulting in around 10 million office visits a year. Diagnosis is often difficult due to its multiple causes—including bacterial vaginosis, vulvovaginal candidiasis, and trichomoniasis—as well as variation in symptom presentation. Typically, cases are identified with a combination of symptomology, medical history, physical examination, and office- or laboratory-based testing. These traditional techniques involve subjective elements and demonstrate varying sensitivity and specificity. Inaccurate or delayed diagnosis leads to continued symptoms, repeat visits, inappropriate treatment, and unnecessary costs. Alternatively, the use of molecular-based assays increases sensitivity for the detection of vaginitis causes. With the validation of the vaginal panel molecular assay on COR (a high-throughput platform), a workflow can be streamlined in high-demand laboratories while providing high sensitivity for vaginitis detection.

## INTRODUCTION

Vaginitis is a common condition among women of reproductive age, resulting in approximately 10 million office visits each year (1–4). Over 70% of vaginitis cases are caused by bacterial vaginosis (BV), vulvovaginal candidiasis (VVC), or trichomoniasis (5). When left untreated, vaginitis can result in pelvic discomfort, pain during intercourse, and other organism-specific complications. In addition, vaginitis can lead to an increased risk of HIV and other sexually transmitted infections and may also lead to complications during pregnancy and pre-term labor (6–11). However, all three vaginitis causative agents can be treated effectively following an accurate diagnosis (8, 12).

Symptoms include abnormal vaginal discharge, odor, irritation, vulvovaginal pruritus or burning, and dyspareunia (13, 14). Although symptom presentation differs among patients, symptoms are typically not specific enough to clearly establish the causal vaginitis agents. Instead, a combination of symptomology, medical history, physical examination, and some form of office- or laboratory-based testing is required (15, 16). Office- or laboratory-based testing facilitates the identification of specific vaginitis causes. BV represents the largest group of infectious vaginitis cases (40%–50%), and testing for BV includes microscopy following normal saline preparation of the specimen and implementation of Amsel’s criteria or, rarely, Gram staining, for research purposes (12, 13, 17, 18). VVC is responsible for approximately 20%–25% of vaginitis cases and can be identified through microscopy following preparation of the specimen with potassium hydroxide or by the establishment of culture (13, 17–19). Trichomoniasis represents the smallest proportion of vaginitis cases (15%) (13, 17, 18). Although trichomoniasis can be diagnosed through the identification of *Trichomonas vaginalis* (TV) through microscopy following wet mount or culture (13), the Centers for Disease Control and Prevention recommends the use of nucleic acid amplification tests (NAATs) for the detection of TV (12). An accurate vaginitis diagnosis has traditionally required specialized in-office microscopy (and related) equipment and staff training (8). However, many real-world vaginitis diagnoses are empirical (20), and less than half of all patient management is based on objective assays (21). For this reason, several NAATs have been developed to provide a means of objective vaginitis diagnosis (22).

The BD MAX Vaginal Panel for BD MAX System (VP-MAX; Becton, Dickinson and Company; BD Life Sciences— Diagnostic Solutions, Sparks, MD, USA) is a multiplex NAAT (real-time PCR-based assay) for specific VVC and TV DNA targets. For BV detection, the vaginal panel includes DNA targets that are followed by fluorogenic, target-specific probes to differentially detect BV markers (23–25). Several studies have demonstrated the analytical and clinical performance of VP-MAX. During a registrational trial, VP-MAX demonstrated statistically better sensitivity for all three vaginitis causes when compared to clinician diagnosis or traditional in-clinic testing (24, 25). These findings were subsequently supported by real-world evidence demonstrating that VP-MAX identifies more cases of vaginitis than clinician diagnosis (26).

The BD COR System (COR; Becton, Dickinson and Company; BD Life Sciences— Diagnostic Solutions, Sparks, MD, USA) is an automated molecular diagnostic system that facilitates high-throughput clinical testing, with minimal user intervention, starting at the pre-analytical specimen handling stage to results reporting. COR consists of a central PX module, which performs all pre-analytical steps, and transitions specimens to either a GX module (for HPV testing) or an MX module (accommodating MAX assays), or a combination thereof. Characterization of COR PX, GX, and MX modules has been previously described for the BD Onclarity HPV assay and the BD CTGCTV2 assay (27, 28). The objective of this study was to compare the performance of the BD Vaginal Panel for BD COR System (VP-COR) to that of VP-MAX and determine whether VP-COR performance meets the criteria for equivalency with VP-MAX.

## MATERIALS AND METHODS

### Study specimens

Clinical specimens were collected under Ethics Committee-approved protocols and were screened for inclusion in this study. Acceptable specimens were pooled by target type to create panel members; when necessary, spiking with a high positive clinical sample or pooling positive samples at target concentrations was performed for the *Candida* group (*C*. group, including *Candida albicans*, *Candida tropicalis*, *Candida parapsilosis*, and *Candida dubliniensis*) and TV panels. For *Candida glabrata* and *Candida krusei*, contrived specimens were created by spiking organisms into negative vaginal matrix due to low prevalence. For BV contrived specimens, simulated vaginal matrix was spiked with known amounts of organisms using quantitated stocks to create panel members with different BV marker combinations. Specimens were prepared according to an expected level and were confirmed as actual levels. Additionally, the *C*. group-positive, TV-positive, and negative vaginitis panel members were analyzed for BV clinical targets. Specimen levels were characterized as one of the following, according to communication from the FDA during the design of this study: (i) negative (negative result expected 100% of the time), (ii) high negative (close to the limit of detection and a positive result 20%–80% of the time), (iii) low positive [close to the limit of detection and a positive result expected 95% of the time (1XC95)], (iv) moderate positive [expected to be positive 100% of the time (3XC95)], and (v) high positive [expected to be positive 100% of the time (covering ranges above 3XC95)]. The distribution of the positive specimens by expected concentration was 60%–80% low or moderate positive with the other 20%–40% high positives. The limit-of-detection values for each of the vaginal panel targets are listed in the package inserts for both the MAX and COR platforms (23, 29).

**Fig 1:**
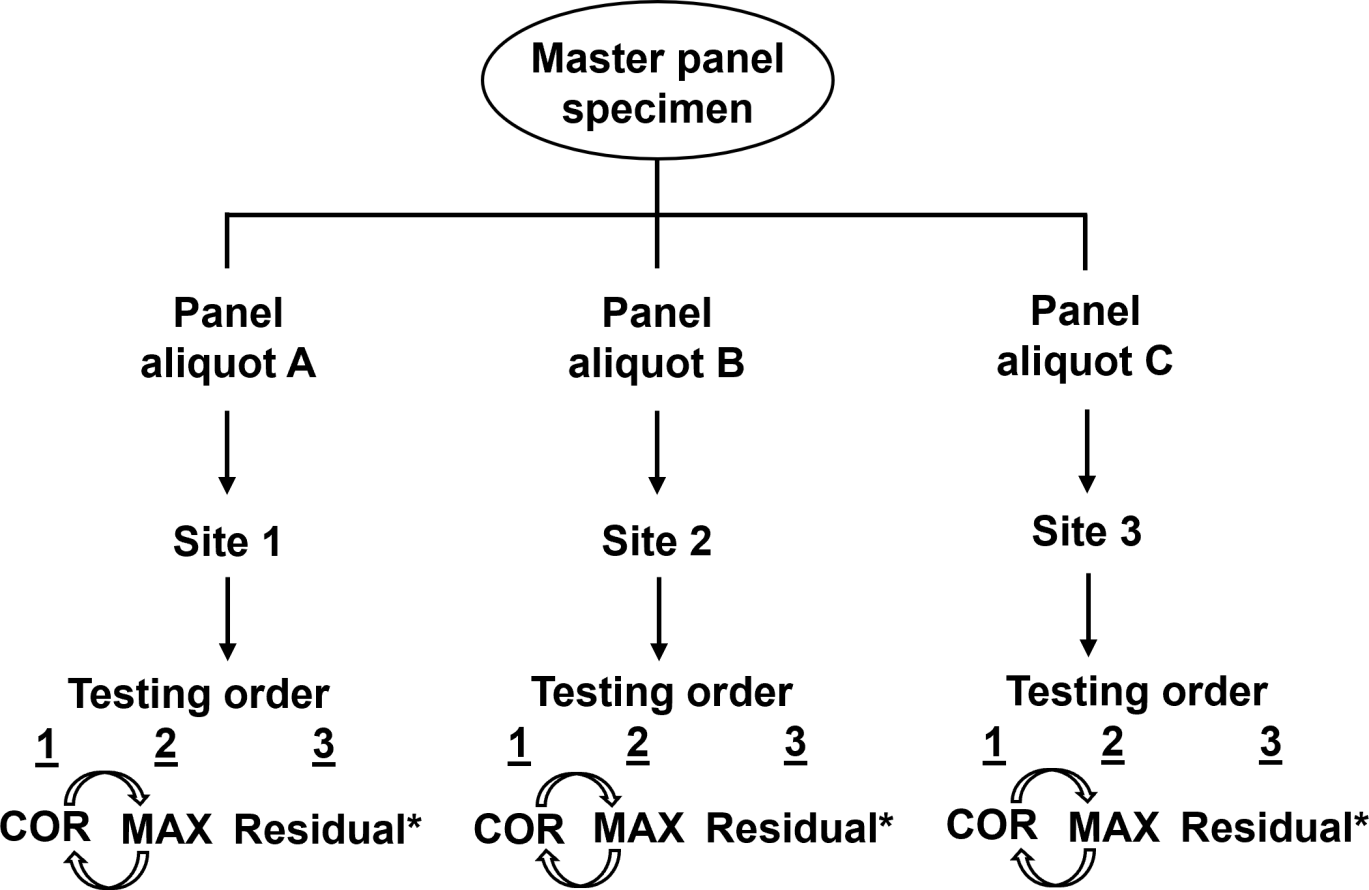
Specimen panel preparation and workflow for the study. Three identical panel sets (A, B, and C) were generated and shipped to the test sites. At the sites, each master panel was utilized for one run on MAX, COR, and a third (see * in figure) for any required repeat testing (see Materials and Methods for details on testing). COR and MAX testing order was randomized for each panel member (indicated by curved arrows).

There were three laboratory testing sites for this study; each had both MAX and COR instruments onsite. The panels were randomized such that each site would test approximately 650 panels with each panel member tested three times for VP-MAX and three times for VP-COR; three replicates of each panel member were tested for each of the three runs for VP-MAX and VP-COR. Paired lot(s) of VP-COR and VP-MAX reagents were used for the study execution (Fig. 1).

### Vaginal panel assay on MAX and COR platforms

VP-MAX (FDA-authorized in October 2016) and VP-COR (FDA-cleared in March 2023) leverage the same real-time PCR reagents for amplification of target DNA, with subsequent tagging using fluorogenic probes to differentially detect BV markers including *Lactobacillus* spp. (*Lactobacillus crispatus* and *Lactobacillus jensenii*), *Atopobium vaginae*, bacterial vaginosis-associated bacteria-2 (BVAB-2), *Gardnerella vaginalis*, and *Megasphaera*-1. A proprietary algorithm is implemented to establish a positive or negative result, based on the concentration of each of the markers stated above. Test positivity for *C*. group, *C. glabrata*, *C. krusei*, and TV was determined by the presence or absence of target DNA (results reported as positive or negative). All processes were automatically handled on MAX and COR platforms, including DNA extraction, reagent rehydration, target amplification, and detection of target nucleic acid sequences.

### Acceptance criteria and statistical analysis

Positive percent agreement (PPA; [index and comparator positive]/[total comparator positive]), negative percent agreement (NPA; [index and comparator negative]/[total comparator negative]), and overall percent agreement (OPA; [(index and comparator positive) + (index and comparator negative)]/[total comparator results]) for VP-COR versus VP-MAX were the major outcomes of interest in this study. The PPA acceptance criteria for each of the vaginitis targets were as follows: BV contrived, point estimate ≥95% with a lower bound of the 95% confidence interval (95% CI) ≥90%; BV clinical, point estimate ≥95%; and *C*. group, *C. glabrata*, *C. krusei*, and TV, point estimate ≥95% with a lower bound of the 95% CI ≥90%. The NPA acceptance criteria for each of the vaginitis targets were as follows: BV contrived, point estimate ≥98%; BV clinical, point estimate ≥95%; and *C*. group, *C. glabrata*, *C. krusei*, and TV, point estimate ≥95% with a lower bound of the 95% CI ≥90%. The non-reportable rate was calculated for VP-MAX and VP-COR. Non-reportable included unresolved (sample or reagent failure), indeterminate (system failure), incomplete run, or external control failure. The sample size was determined by calculating the margin of error for a score confidence interval to fall below 5%. Here, the margin of error for 150 samples (for the three sites, combined) was <4.8% and for 300 samples (for the three sites, combined) was <3.1%. Point estimates with Clopper–Pearson confidence intervals were calculated according to Altman (30).

## RESULTS

Specimen panels were prepared at concentrations including negative, high negative (BV clinical, *C*. group, and TV only), low positive, moderate positive, and high positive for BV, VVC, and trichomoniasis causes associated with vaginitis. The panels used for VP-MAX and VP-COR testing are listed according to the final VP-MAX result concentration and number for each of the targets (Table 1). The total numbers tested were 516 BV contrived, 1,055 BV clinical, 544 *C*. *glabrata*, 522 *C*. *krusei*, 724 *C*. group, and 702 TV.

**TABLE 1:** Panel results by VP-MAX^*a*^ (comparator) result concentration and number for each target^*b*^

Specimen category	Final MAX level					Final result (2 of 3 VP-MAX results)	Number
	N	HN	LP	MP	HP		
BV contrived	300	0	0	0	0	Negative	300
	0	0	77	139	0	Positive	216
							516
BV clinical	330	25	3	0	0	Negative	358
	3	4	420	270	0	Positive	697
							1,055
*C. glabrata*	372	0	0	0	0	Negative	372
	0	0	62	71	39	Positive	172
							544
*C. krusei*	372	0	0	0	0	Negative	372
	0	0	59	59	32	Positive	150
							522
*C.* group	372	0	0	0	0	Negative	372
	0	36	111	120	85	Positive	352
							724
TV	372	0	0	0	0	Negative	372
	3	16	89	105	117	Positive	330
							702

^*a*^ The MAX grouping includes both reportable and non-reportable results.

^*b*^ VP-MAX, vaginal panel for MAX platform; N, negative; HN, high negative; LP, low positive; MP, moderate positive; HP, high positive; BV, bacterial vaginosis; TV, *Trichomonas vaginalis*.

PPA, NPA, and OPA values for VP-COR, when compared to VP-MAX, were calculated based on the results in Table 2. For BV contrived and BV clinical specimens, VP-COR had a PPA with VP-MAX of 99.5% [97.5, 100] and 97.9% [96.5, 98.8], respectively. NPA associated with VP-COR for BV contrived and BV clinical specimens was 100% [98.8, 100] and 95.8% [93.2, 97.6], respectively. For *C. glabrata*, *C. krusei*, and *C*. group, the PPA for VP-COR compared to VP-MAX was 100% [97.9, 100], 100% [97.6, 100], and 99.4% [98.0, 99.9], respectively; NPA was 100% [99.0, 100], 100% [99.0, 100], and 98.9% [97.3, 99.7], respectively. For TV detection, the PPA and NPA values, respectively, were 99.7% [98.3, 100] and 100% [99.0, 100].

**TABLE 2:** VP-COR versus VP-MAX performance for vaginitis detection^*a*^

	PPA [95% CI] (*n*/*N*)	NPA [95% CI] (*n*/*N*)	OPA [95% CI] (*n*/*N*)
BV contrived	99.5% [97.5, 100] (215/216)	100% [98.8, 100] (300/300)	99.8% [98.9, 100] (515/516)
BV clinical	97.9% [96.5, 98.8] (682/697)	95.8% [93.2, 97.6] (343/358)	97.2% [96.0, 98.1] (1,025/1,055)
*C. glabrata*	100% [97.9, 100] (172/172)	100% [99.0, 100] (372/372)	100% [99.3, 100] (544/544)
*C. krusei*	100% [97.6, 100] (150/150)	100% [99.0, 100] (372/372)	100% [99.3, 100] (522/522)
*C.* group	99.4% [98.0, 99.9] (350/352)	98.9% [97.3, 99.7] (368/372)	99.2% [98.2, 99.7] (718/724)
TV	99.7% [98.3, 100] (329/330)	100% [99.0, 100] (372/372)	99.9% [99.2, 100] (701/702)

^*a*^ VP-COR, vaginal panel for COR platform; VP-MAX, vaginal panel for MAX platform; PPA, positive percent agreement; NPA, negative percent agreement; OPA, overall percent agreement; BV, bacterial vaginosis, TV, *Trichomonas vaginalis*.

Within the BV contrived group, there was one false negative result for COR (with MAX as comparator) that was a moderate positive according to the final MAX level (Table 3). For BV clinical, there were 15 false negative results for COR that included 2 high negative, 1 negative, 10 low positive, and 2 moderate positive specimens by the final MAX level; there were 15 false positive results that included 8 high negative and 7 negative specimens. For *C. glabrata*, there were two false negative results for COR that were both low positives by the final MAX level and four false results that were negative. For TV, there was one false negative result for COR that was a low positive by the final MAX level (Table 3).

**TABLE 3:** Final MAX level according to MAX versus COR discordant results^*a*^

Specimen type	MAX result	COR result	Final MAX level	*n*	Group discordant rate	Overall discordant rate
BV contrived (*n* = 516)	Positive	Negative	Moderate positive	1	0.2%	0.2%
BV clinical (*n* = 1,055)	Positive	Negative	Low positive	10	0.9%	2.8%
	Positive	Negative	High negative	2	0.1%	
	Positive	Negative	Negative	1	0.2%	
	Positive	Negative	Moderate positive	2	0.2%	
	Negative	Positive	High negative	8	0.8%	
	Negative	Positive	Negative	7	0.7%	
*C. glabrata* (*n* = 544)	Positive	Negative	Low positive	2	0.4%	1.1%
	Negative	Positive	Negative	4	0.7%	
TV (*n* = 702)	Positive	Negative	Low positive	1	0.1%	0.1%

^*a*^ BV, bacterial vaginosis; TV, *Trichomonas vaginalis*.

Initial non-reportable rates for VP-MAX and VP-COR were 0.92% [0.56, 1.44] and 0.64% [0.34, 1.09], respectively (Table 4). Following repeat testing, the VP-MAX non-reportable rate fell to 0.05% [0.00, 0.27], while the VP-COR rate fell to 0.00% [0.00, 0.20].

**TABLE 4:** Non-reportable rates for VP-COR and VP-MAX^*a*^

Platform	Total UNR/IND/INC rates	
	Initial rate [95% CI] (*n*/*N*)	Final rate [95% CI] (*n*/*N*)
MAX	0.92% [0.56, 1.44] (19/2,055)	0.05% [0.00, 0.27] (1/2,054)
COR	0.64% [0.34, 1.09] (13/2,047)	0.00% [0.00, 0.20] (0/2,044)

^*a*^ UNR, unresolved rate; IND, indeterminate; INC, incomplete.

## DISCUSSION

This study was a comparison between VP-COR and VP-MAX, based on data from the registrational study to obtain an indication for vaginal panel performance on the COR instrument. Compared to the VP-MAX (comparator), VP-COR (index) showed high PPA, NPA, and OPA for all of the target organisms. For BV contrived and clinical specimens, the PPA point estimates were both ≥95% (and the 95% confidence interval lower bound value for BV contrived was ≥90%); for BV contrived, the NPA point estimate was 100% (which met the criteria of ≥98%), and for BV clinical, the NPA point estimate was 95.8% (which met the criteria of ≥95%). The PPA and NPA point estimates for *C. glabrata* (100% and 100%, respectively), *C. krusei* (100% and 100%, respectively), and *C*. group (99.4% and 98.9%, respectively) were all ≥95% (and all three had 95% confidence interval lower bound values ≥90% for both performance outcomes). Finally, for TV, VP-COR had a PPA point estimate of 99.7% (which met the criteria of ≥95%) with a 95% confidence interval lower bound value of 98.3% (which met the criteria of ≥90%) and an NPA point estimate of 100% (which met the criteria of ≥95%) with a 95% confidence interval lower bound value of 99.0% (which met the criteria of ≥90%). The non-reportable rates for VP-MAX and VP-COR were both <1%. As expected, the BV clinical group had the highest overall discordant rate (2.8%) as that specimen type was not spiked or prepared as a panel according to the target level. However, the false positive and false negative rates were low for COR, and COR had a high overall percent agreement with MAX.

Both registrational and real-world studies have recently demonstrated the clinical efficacy of the vaginal panel assay for the diagnosis of vaginitis on the MAX platform. However, MAX is a benchtop instrument, typically utilized for local testing. Health care providers, such as primary care physicians, often have testing performed at centralized laboratories for specimens collected in-clinic. COR is currently the only high-throughput platform on the market with full pre-analytical capacity (PX module) in combination with an integrated analytic system (MX module) in one instrument. The latter is important because the pre-analytical workflow often accounts for a large proportion of staff hands-on time at centralized laboratories. Therefore, VP-COR provides excellent performance for vaginitis detection while streamlining workflow in high-demand laboratories.

### Limitations

This study included some BV panel members that were not clinical specimens. These contrived BV specimens consisted of the organisms that make up the vaginal panel BV grouping, spiked into simulated vaginal matrix at different ratios to represent low positive, moderate positive, and high positive clinical specimens. Therefore, these specimens were not the ideal challenge specimen types for determining the clinical performance of VP-COR.

### Conclusions

VP-COR met the acceptance criteria to establish equivalency with VP-MAX when tested across all target levels.
